# Connexin 43 Hemichannel as a Novel Mediator of Sterile and Infectious Inflammatory Diseases

**DOI:** 10.1038/s41598-017-18452-1

**Published:** 2018-01-09

**Authors:** Wei Li, Guoqiang Bao, Weiqiang Chen, Xiaoling Qiang, Shu Zhu, Shuaiwei Wang, Mingzhu He, Gaifeng Ma, Mahendar Ochani, Yousef Al-Abed, Huan Yang, Kevin J. Tracey, Ping Wang, John D’Angelo, Haichao Wang

**Affiliations:** 1 Department of Emergency Medicine, North Shore University Hospital, Northwell Health, Manhasset, NY 11030 USA; 2 The Feinstein Institute for Medical Research, 350 Community Drive, Manhasset, NY 11030 USA; 3 International Laboratory for Sepsis Research, Huaihe Hospital, Henan University, Kaifeng, Henan 475000 China; 4 Department of General Surgery, Tangdu Hospital, The 4th Military Medical University, Xi’an, Shaanxi 710032 China

## Abstract

Cytoplasmic membrane-bound connexin 43 (Cx43) proteins oligomerize into hexameric channels (hemichannels) that can sometimes dock with hemichannels on adjacent cells to form gap junctional (GJ) channels. However, the possible role of Cx43 hemichannels in sterile and infectious inflammatory diseases has not been adequately defined due to the lack of selective interventions. Here we report that a proinflammatory mediator, the serum amyloid A (SAA), resembled bacterial endotoxin by stimulating macrophages to up-regulate Cx43 expression and double-stranded RNA-activated protein kinase R (PKR) phosphorylation in a TLR4-dependent fashion. Two well-known Cx43 mimetic peptides, the GAP26 and TAT-GAP19, divergently affected macrophage hemichannel activities *in vitro*, and differentially altered the outcome of lethal sepsis *in vivo*. By screening a panel of Cx43 mimetic peptides, we discovered that one cysteine-containing peptide, P5 (ENVCYD), effectively attenuated hemichannel activities, and significantly suppressed endotoxin-induced release of ATP and HMGB1 *in vitro*. *In vivo*, the P5 peptide conferred a significant protection against hepatic ischemia/reperfusion injury and lethal microbial infection. Collectively, these findings have suggested a pathogenic role of Cx43 hemichannels in sterile injurious as well as infectious inflammatory diseases possibly through facilitating extracellular ATP efflux to trigger PKR phosphorylation/activation.

## Introduction

Following injury and infection, innate immune cells (such as macrophages and monocytes) mount an immediate inflammatory response by releasing a wide array of early proinflammatory cytokines (e.g., TNF, IL-1, and IFN-γ). Some early cytokines also modulate the expression of a liver-derived acute phase protein, the serum amyloid A (SAA)^1^, which subsequently signals via the receptor for the advanced glycation end products (RAGE)^2^, TLR2^3^, TLR4^4^ or P2X_7_ receptor (P2X_7_R)^5^ to further induce various cytokines and chemokines. Meanwhile, exogenous microbial toxins (e.g., endotoxin)^6^ and endogenous proinflammatory cytokines [e.g., IFN-γ, cold-inducible RNA-binding protein **(**CIRP), or the SAA]^7–9^ also stimulate macrophages/monocytes to secrete other “late” inflammatory mediators such as the high mobility group box 1 (HMGB1). The extracellular release of HMGB1 is partly regulated by the double-stranded RNA-activated protein kinase R (PKR)-mediated inflammasome activation and pyroptosis^10^, a type of cell death characterized by a rapid plasma membrane rupture and release of cellular inflammatory contents (including HMGB1). Indeed, the genetic disruption of PKR expression or pharmacological inhibition of its phosphorylation similarly impairs inflammasome activation^10,11^, pyroptosis^10,11^, and the resultant HMGB1 release^10^.

In addition, HMGB1 can be passively released from damaged cells^12^ following ischemia/reperfusion (I/R) injury^13^, thereby serving as a damage-associated molecular pattern molecule (DAMP). When released excessively, the extracellular HMGB1 adversely contributes to the pathogenesis of injury- or infection-elicited inflammatory diseases^14^, because HMGB1-neutralizing antibodies conferred a significant protection against I/R injury^13^, lethal endotoxemia^6^, and sepsis^15,16^. Thus, infection and injury converge on a common process – inflammation^14^, which is orchestrated by HMGB1 and other proinflammatory mediators (e.g., mitochondrial DNA and CIRP) released by activated innate immune cells and damaged tissues^8,17^.

In parallel, bacterial endotoxins and proinflammatory cytokines (e.g., TNF and IFN-γ) also up-regulated the expression of Connexin 43 (Cx43) in macrophage/monocyte cultures^18,19^. Cx43 carries four trans-membrane domains that embrace two extracellular loops (EL), one intracellular loop, as well as the intracellular N- and C-termini^20^. The cytoplasmic membrane-bound Cx43 oligomerize to form hexameric hemichannels^21^, which can dock with the hemichannels on adjacent cells to form GJ channels particularly in non-immune cells (of the heart, brain and vasculature) to facilitate intercellular communications^22–24^. However, it was previously unknown whether Cx43 hemichannel occupies a possible role in the pathogenesis of injury- and infection-elicited excessive inflammation due to lack of selective interventions.

The establishment of HMGB1 as a critical mediator of various inflammatory diseases has prompted an on-going search for endogenous and exogenous modulators of HMGB1 secretion. For instance, we discovered that a chemical derivative of the major Gancao (*Radix glycyrrhizae*) component (glycyrrhizin or glycyrrhizic acid, GZA), termed the carbenoxolone (CBX), effectively prevented the endotoxin-induced PKR phosphorylation and the PKR-mediated HMGB1 secretion^25^. Given the CBX’s capacity in blocking Cx43 GJ and hemichannel activities, we reasoned that macrophage Cx43 hemichannels might also be involved in the regulation of HMGB1, a key mediator of injury- and infection-elicited inflammatory diseases.

In this study, we sought to examine whether SAA up-regulates the expression of Cx43 in innate immune cells, and whether interventions of Cx43 hemichannels influence the outcome of lethal sepsis. Furthermore, we developed a class of novel mimetic peptides (P5) that selectively inhibited macrophage hemichannel activities without impairing GJ communications, and further explored their therapeutic potential in animal models of hepatic I/R injury and lethal sepsis.

## Results

### Exogenous endotoxin and endogenous proinflammatory mediator (SAA) induced parallel Cx43 upregulation and PKR phosphorylation

**Figure 1:**
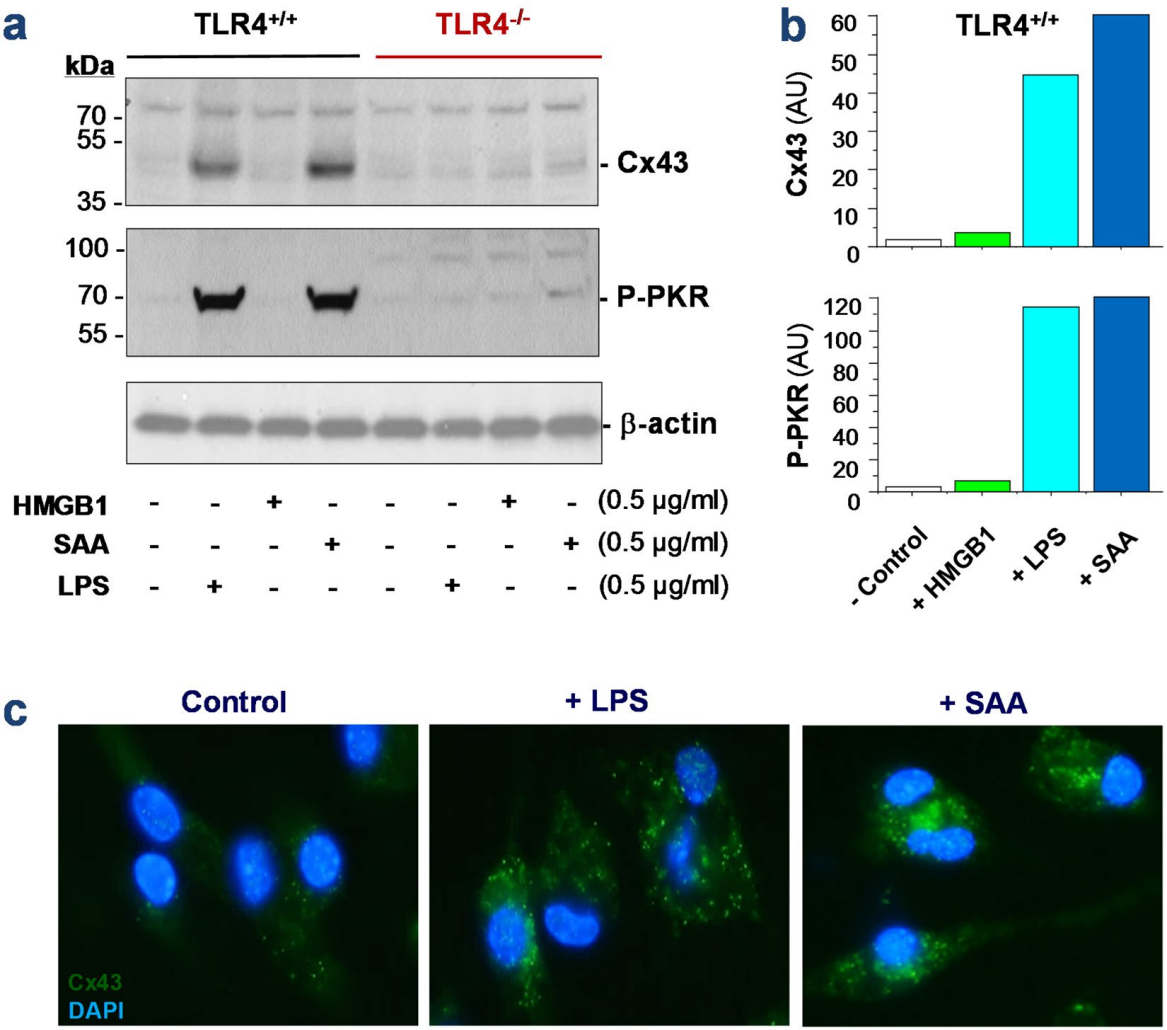
Exogenous bacterial endotoxin (LPS) and endogenous inflammatory cytokines (SAA) up-regulated Cx43 expression and PKR phosphorylation in macrophages. Primary murine peritoneal macrophages were isolated from wild-type or TLR4-deficient C57BL/6 mice, and stimulated with crude LPS, recombinant HMGB1, or SAA for 16 h. The cellular levels of Cx43 and phospho-PKR (P-PKR) were measured by Western blotting (Panel a,b) or immunocytochemistry (Panel c), respectively.

To assess the possible role of Cx43 hemichannel in the regulation of innate immunity, we measured the Cx43 expression in wild-type and TLR4-deficient peritoneal macrophages after prolonged (16 h) stimulation with bacterial endotoxin or SAA. Although quiescent macrophages constitutively expressed Cx43 at relative low levels (Fig. 1a–c), crude LPS and SAA, but not HMGB1, induced a marked elevation of Cx43 expression (by 45–60 folds) in the wild-type, but not in the TLR4-deficient macrophages (Fig. 1a), suggesting that LPS and SAA induced Cx43 expression in a TLR4-dependent fashion. Consistent with our previous report^9^, the crude LPS and purified SAA induced a parallel and marked phosphorylation of PKR (Fig. 1a), a recently identified key regulator of HMGB1 release in innate immune cells^10^. Similarly, the marked elevation of PKR phosphorylation occurred only in the wild-type, but not in the TLR4-deficient macrophages (Fig. 1a), confirming an essential role of TLR4 in the LPS/SAA-induced PKR phosphorylation. In contrast, the deletion of TLR2 did not affect the LPS- or SAA-induced Cx43 upregulation and PKR phosphorylation (data not shown), suggesting an essential role for TLR4, but not TLR2, in the upregulation of Cx43 expression and PKR phosphorylation in activated macrophages.

### Divergent impact of Cx43 mimetic peptides on macrophage hemichannel activities and survival of lethal sepsis

**Figure 2:**
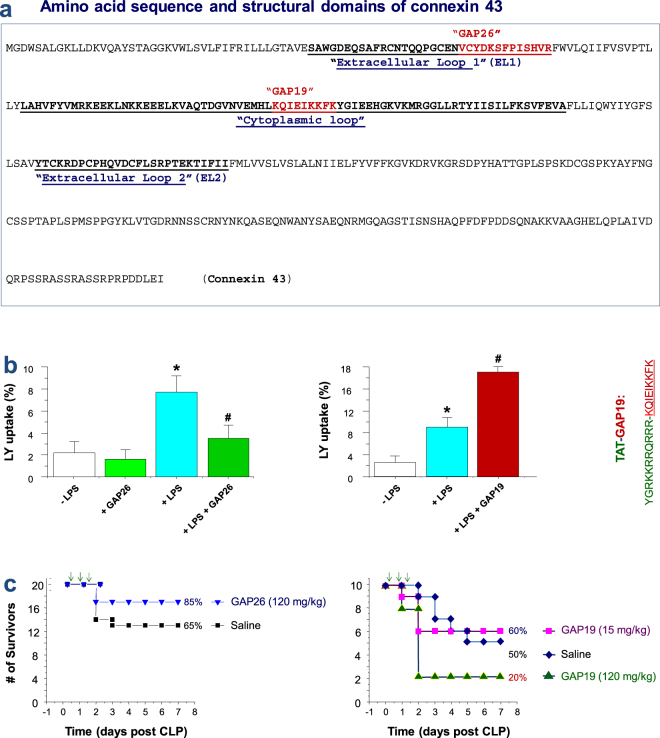
Cx43 mimetic peptides GAP26 and GAP19 divergently affected macrophage hemichannel activities and septic lethality. (**a**) Amino acid sequence and membrane topology of Cx43. The relative localizations and sequences of two  mimetic peptides, GAP26 and GAP19 were indicated. (**b**) GAP26 and GAP19 divergently affect macrophage hemichannel activities. RAW 264.7 cells were stimulated with LPS (0.5 μg/ml) in the absence or presence of GAP26 (100 μM) or TAT-GAP19 (200 μM) for 16 h, and subsequently incubated with Lucifer Yellow (LY, 1 mg/ml) for 15 min. After fixation and three extensive washes, the number of cells with diffused fluorescent signals was counted under a fluorescence microscope, and expressed as a percentage of total cell numbers (DAPI-stained nuclei) in six fields. *P < 0.05 versus “−LPS”; ^#^ *P* < 0.05 versus “+LPS”. (**c**) Divergent effects of GAP26 and TAT-GAP19 on septic animal lethality. Balb/c mice were subjected to CLP-induced sepsis, and intraperitoneally administered with saline, GAP26 or TAT-GAP19 at +6, +18 and +36 h post CLP. Animal survival rates were monitored for two weeks and  no later death occurred.

The up-regulated Cx43 expression may lead to enhanced hemichannel (but not GJ) activities, as macrophages do not form GJ between themselves^25,26^. We thus evaluated the alteration of macrophage hemichannel activities in the absence or presence of Cx43 mimetic peptides previously shown to be capable of blocking Cx43 hemichannels. For instance, GAP26 mimics a short stretch of amino acids on the first extracellular loop (EL1, Fig. 2a), and may interact with yet-undefined sequence on the extracellular loops of the Cx43, thereby inhibiting Cx43 hemichannel activities or gap junction formation^27^. Similarly, a cytoplasmic loop mimetic peptide termed GAP19 has been shown to selectively block Cx43 hemichannel activities in non-immune cells such as the Hela cells, cardiac myocytes and astrocytes^28–30^. Unlike hemichannels formed by pannexin 1 (Panx1), the Cx43 hemichannels are sensitive to calcium (Ca^2+^), and remain closed in the presence of extracellular calcium^31,32^. Indeed, endotoxin stimulation did not significantly increase the number of LY-positive macrophages in the presence of calcium (data not shown), indicating that LPS treatment did not alter Panx1 hemichannel permeability. However, when macrophages were cultured in the calcium-depleted DMEM medium, LPS caused a marked increase in the percentage of LY-positive cells (Fig. 2b), suggesting an elevation of Cx43 hemichannel activities. As expected, the pre-incubation of macrophages with the GAP26 peptide resulted in a significant impairment of the endotoxin-induced elevation of hemichannel activities (Fig. 2b, left panel). In a sharp contrast, the TAT-GAP19 surprisingly elevated LY dye uptake (Fig. 2b, right panel), suggesting that TAT-GAP19 adversely increased macrophage hemichannel activities.

Given the pathogenic roles of PKR in sepsis^10^ and the  association between PKR phosphorylation and Cx43 expression in macrophages, we assessed the impact of these Cx43 mimetic peptides on the outcome of a clinically relevant animal model of lethal sepsis induced by cecal ligation and puncture (CLP). As expected, GAP26 peptide reproducibly increased animal survival rates (Fig. 2c, left panel). In contrast, TAT-GAP19 decreased animal survival particularly when given at a comparable higher dose (Fig. 2c). Thus, the distinct effects of GAP26 and TAT-GAP19 on the macrophage hemichannel activities correlated to their divergent impact on the outcome of lethal sepsis, suggesting that the excessive Cx43 hemichannel activation may contribute to the pathogenesis of lethal systemic inflammation.

### Development of selective macrophage Cx43 hemichannel inhibitors preserving GJ function in non-immune cells

**Figure 3:**
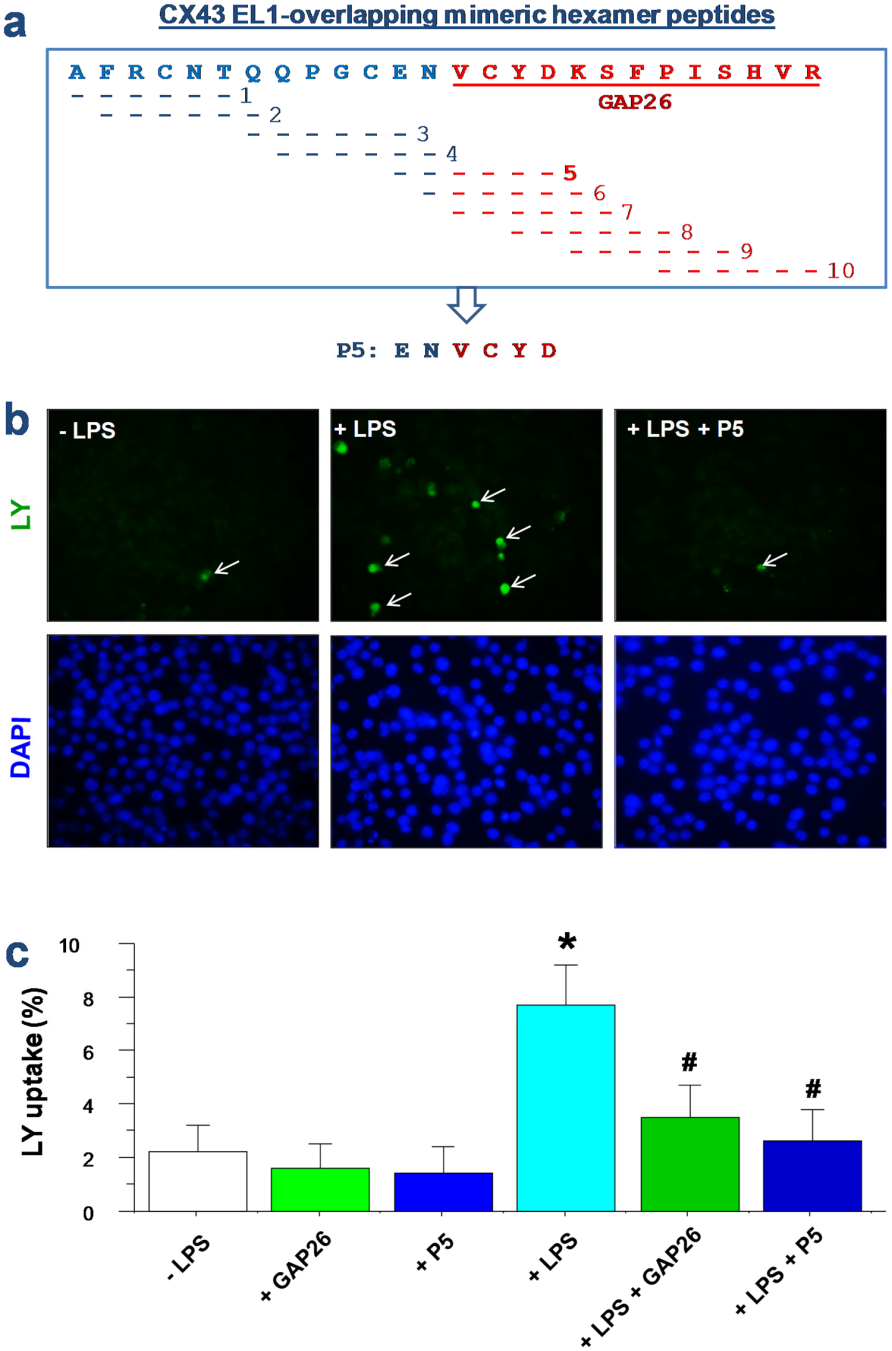
Design of a class of Cx43 mimetic peptides capable of selectively inhibiting macrophage hemichannel activities. (**a**) Sequence of Cx43 mimetic peptides. A panel of ten smaller peptides corresponding to the extracellular loop 1 (EL1) of Cx43 were synthesized, and screened for activities in inhibiting macrophage hemichannel activities. (**b**,**c**) P5 significantly inhibited the LPS-induced elevation of Lucifer Yellow (LY) dye uptake. RAW 264.7 cells were stimulated with LPS (0.5 μg/ml) in the absence or presence of Cx43 peptide antagonists (GAP26, 100 μM; or P5, 20 μM) for 16 h, and subsequently incubated with LY (1 mg/ml) for 15 min. After fixation and three extensive washes, the number of cells with diffused fluorescent signals was counted under a fluorescence microscope (Panel b), and expressed as a percentage of total cell numbers (DAPI-stained nuclei) in six fields (Panel c). *P < 0.05 versus “−LPS”; ^#^ *P* < 0.05 versus “+LPS”.

To further elucidate the possible role of Cx43 hemichannels in inflammatory diseases, we synthesized a panel of Cx43 EL1-overlapping hexamer peptides (Fig. 3a), and screened each for potential activities in modulating macrophage hemichannel activities. One of the cysteine-containing peptide, P5, significantly inhibited the endotoxin-induced elevation of hemichannel activities, as manifested by the significant decrease in the cellular LY uptake (Fig. 3b,c).

**Figure 4:**
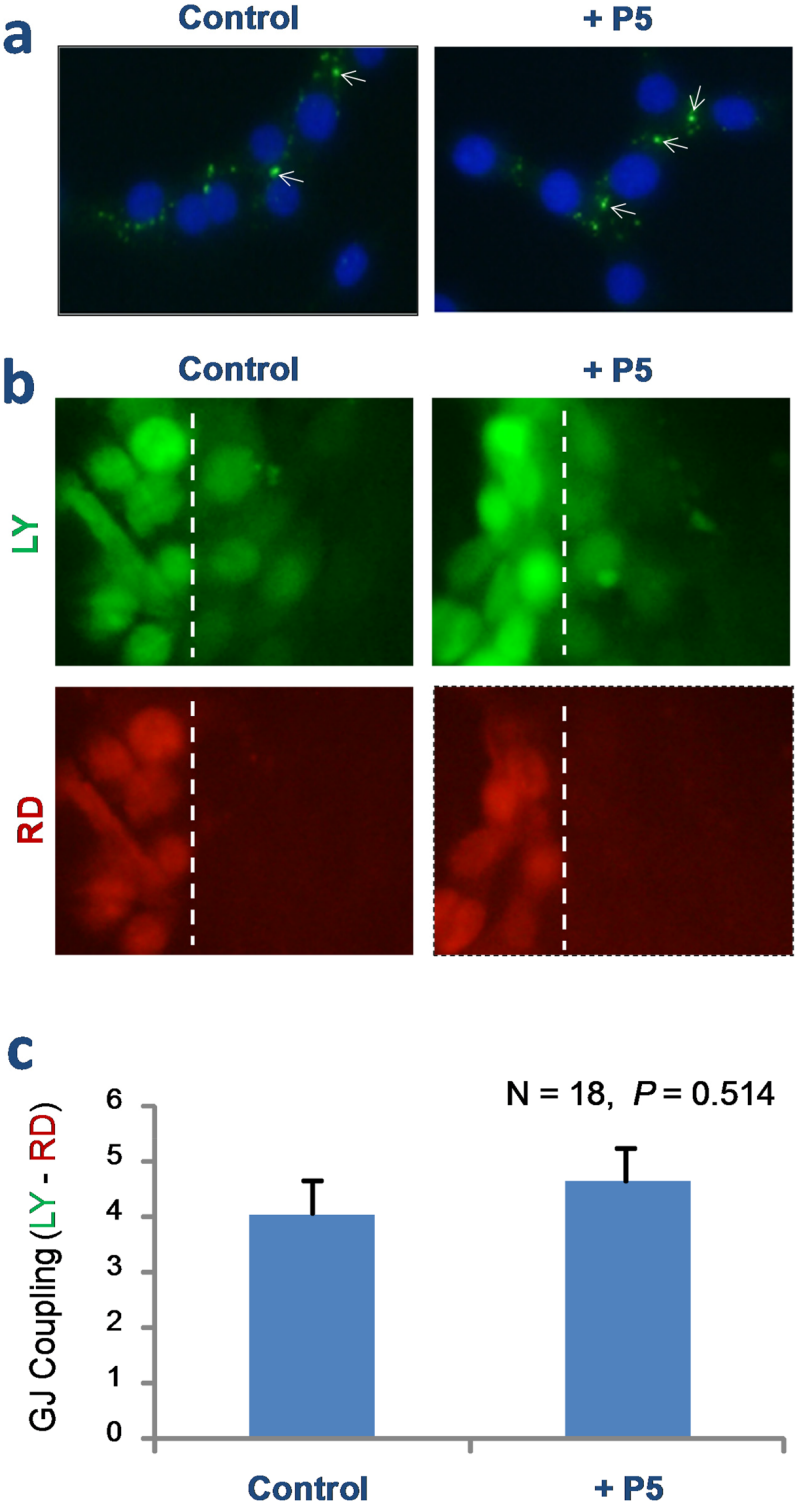
P5 peptide did not impair Cx43 gap junction activities. (**a**) Immunocytochemical analysis of Cx43 gap junction formation in NIH 3T3 fibroblasts. Overnight incubation of NIH 3T3 cells with P5 peptide did not alter the punctate immunostaining profile of Cx43 (arrows), a typical characteristics of Cx43 gap junctions. (**b**,**c**) Scrape-loading assay of GJ intercellular communication. Following the scrape-loading of a low (444 Da, LY) and high (10,000 Da, RD) fluorescent dyes, the transfer of these fluorescent dyes into contiguous cells was monitored. Note that P5 peptide did not affect the gap junctional transfer of LY. Dotted lines indicated the position of pipette tip scrape loading.

Cx43 hemichannels can dock with the hemichannels on adjacent cells to form GJ channels that facilitate intercellular communications^22–24^. To assess whether the P5 peptide affects GJ function, we examined its effect on Cx43 gap junction formation and gap junctional intercellular communication in Cx43-expressing NIH 3T3 fibroblasts. Even a prolonged incubation of NIH 3T3 cells with P5 peptide did not alter Cx43 protein expression (as judged by Western blotting analysis of cellular proteins, data not shown) or GJ formation as judged by the characteristic intercellular punctate profile of Cx43 immunostaining (arrows, Fig. 4a).

Furthermore, we employed the scrape-loading technique to assess P5’s effect on the GJ-mediated intercellular communication. Following the scrape-loading of a low MW (444 Da) LY into the NIH 3T3 fibroblasts, we observed a quick transfer of LY fluorescence into contiguous cells within minutes (Fig. 4b, LY, top panels). The involvement of GJ in the observed LY dye transfer was verified by the concurrent loading of a higher MW (10,000 Da) rhodamine dextran (RD) dye, which could not cross the relatively narrow GJ when introduced intracellularly by the scrape loading (Fig. 4b, RD, bottom panels). Furthermore, there was no obvious difference in the LY diffusion or RD immobility patterns between the control and P5-treated cells (Fig. 4c), suggesting that P5 did not affect the GJ formation or function in non-immune cells.

### P5 peptide inhibited endotoxin-induced ATP release and trypan blue dye uptake

**Figure 5:**
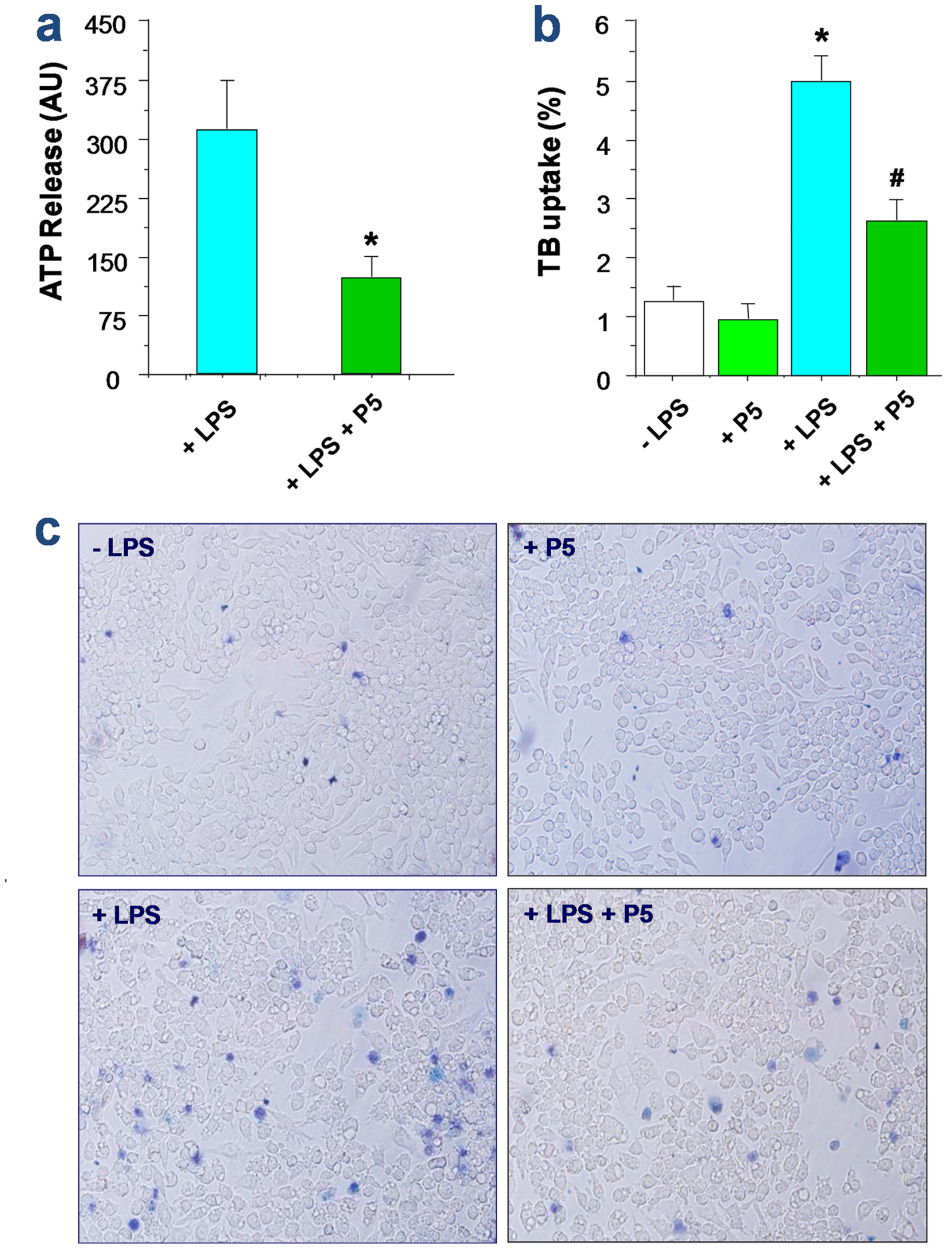
P5 significantly attenuated the LPS-induced ATP release and trypan blue dye uptake in macrophage cultures. (**a**) P5 peptide attenuated the LPS-induced ATP release. *P < 0.05 versus “+LPS”. (**b**,**c**) P5 peptide significantly suppressed the LPS-induced trypan blue dye uptake. RAW 264.7 cells were stimulated with LPS in the absence or presence of P5 peptide for 16 h, and stained with trypan blue dye. Phase contrast images were randomly captured from multiple fields (Panel c), and the percentage of trypan blue-stained cells were calculated (Panel b). *P < 0.05 versus “−LPS”; ^#^ *P* < 0.05 versus “+LPS”.

Because Cx43 hemichannels may provide a mode of ATP release from activated innate immune cells^33,34^, we tested whether the P5 peptide also affected the endotoxin-induced ATP release by macrophage cultures. Although P5 peptide did not affect the LPS-induced Cx43 upregulation (data not shown), it significantly attenuated the LPS-induced ATP release (Fig. 5a). Similarly, it also significantly suppressed the LPS-induced elevation of trypan blue uptake (Fig. 5b,c), suggesting that P5 peptide effectively impaired the LPS-induced elevation of hemichannel activities or the resultant cell death, as judged collectively by the LY dye uptake, ATP release, as well as trypan blue exclusion.

### P5 selectively attenuated the endotoxin-induced HMGB1 release

**Figure 6:**
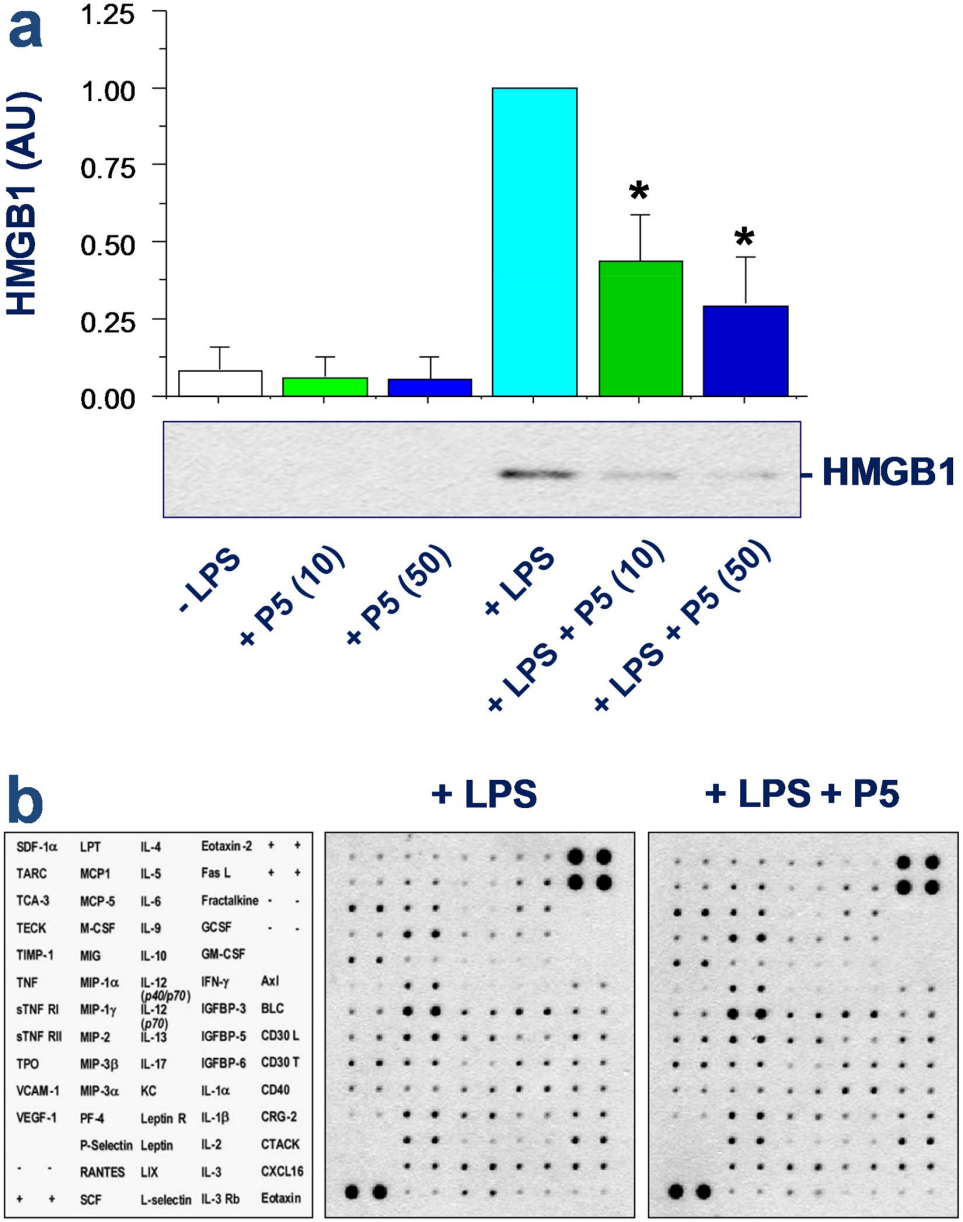
P5 peptide selectively inhibited endotoxin-induced HMGB1 release. RAW 264.7 cells were stimulated with LPS for 16 h in the absence or presence of P5 peptide at indicated concentrations, and the levels of HMGB1 and other cytokines or chemokines in the macrophage-conditioned culture medium were determined by Western blotting analysis (Panel a) or Cytokine Antibody Arrays (Panel b; P5, 10 μM), respectively. *P < 0.05 versus “+LPS”.

Given the possible role of ATP in the regulation of PKR activation and HMGB1 release^25^, we next examined P5’s effects on the LPS-induced HMGB1 release. Prolonged stimulation with the crude LPS (0.5 µg/m1, 16 h) caused a marked increase of extracellular HMGB1 release (Fig. 6a), which was significantly inhibited by P5 in a dose-dependent fashion (Fig. 6a). In contrast, P5 peptide did not affect the LPS-induced production of nitric oxide (data not shown), or other cytokines and chemokines as judged by the cytokine antibody arrays (Fig. 6b). These results suggest a possibility that P5 peptide may selectively inhibit the endotoxin-induced release of HMGB1 by innate immune cells.

### P5 peptide conferred a significant protection against hepatic I/R injury and lethal sepsis

**Figure 7:**
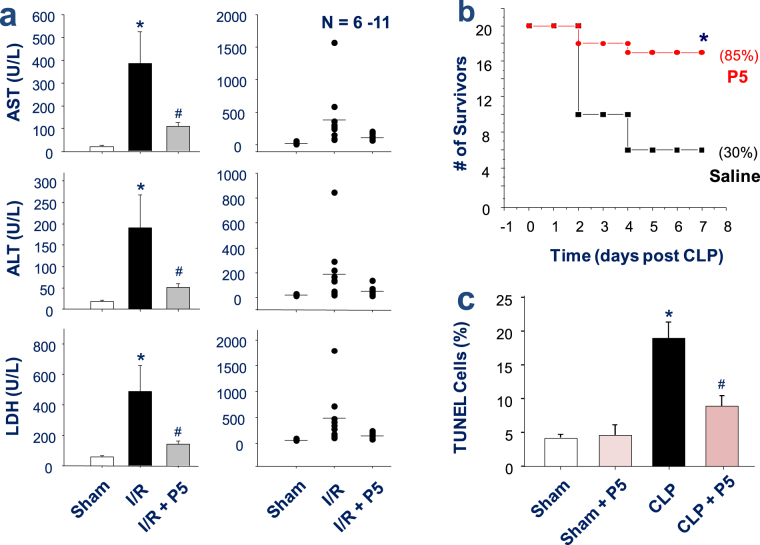
P5 peptide significantly protected mice against hepatic I/R injury and lethal sepsis. (**a**) P5 peptide conferred a significant protection against hepatic I/R injury. Male C57BL/6 mice were subjected to hepatic ischemia and intravenously administered with 0.2 ml saline or P5 peptide solution (10.0 mg/kg BW) at the onset of reperfusion. At 24 h after the onset of ischemia, animals were euthanized to harvest blood to measure serum markers of hepatic injury (e.g., AST and ALT) and LDH. *P < 0.05 versus “Sham control”; ^#^ *P* < 0.05 versus Saline group (“I/R”). (**b**) P5 peptide protected mice against lethal sepsis. Male Balb/c mice were subjected to lethal sepsis by CLP, and intraperitoneally administered with control saline (0.2 ml/mouse) or P5 peptide (8.0 mg/kg) at +2, +24 hours post CLP. Animal survival was assessed for up to two weeks, and the Kaplan-Meier method was used to compare the differences in mortality rates between groups. *P < 0.05 versus “CLP + Saline” group. (**c**) P5 peptide reduced TUNEL-positive staining in the lungs of septic animals. The lungs of septic mice were harvested at 24 h post CLP, and tissue sections were stained by TUNEL staining and DAPI. The number of TUNEL-positive green cells was expressed as  an average percentage of total DAPI-positive blue cells in 5–8 randomly selected fields. *P < 0.05 versus “Sham” control; ^#^ *P* < 0.05 versus “CLP” group.

Given the selective inhibitory effects of P5 on HMGB1 release and the role of HMGB1 in the pathogenesis of both sterile and infectious inflammatory diseases, we investigated the effects of P5 in murine models of hepatic I/R injury and sepsis. As previously reported^35^, the temporal clamping of the hepatic artery and portal vein (for 60 min) produced a marked ischemia in ~70% of the liver (data not shown). The intravenous infusion of P5 peptide (10.0 mg/kg BW), immediately prior to the onset of reperfusion, resulted in a significant reduction of serum levels of hepatic injury markers including the alanine aminotransferase (ALT) and aspartate aminotransferase (AST) (Fig. 7a). Moreover, another marker of cell injury, the systematic release of LDH, was also significantly attenuated by the P5 peptide infusion (Fig. 7a).

In a clinically relevant animal model of polymicrobial sepsis induced by CLP, repetitive administration of P5 (8.0 mg/kg) at +2 h and +24 h *after* the onset of sepsis promoted a significant increase in animal survival rates (Fig. 7b). Together with the effects of GAP26 and GAP19 on hemichannel and sepsis lethality, these results strongly suggest a relationship between Cx43 hemichannel activity and sepsis pathogenesis. TUNEL analysis revealed that lungs of septic mice had a significant increase in the number of TUNEL-positive cells than in sham controls (Fig. 7c). However, at the doses that conferred a significant protection, P5 significantly reduced the number of TUNEL-positive cells in septic lungs (Fig. 7c). Together with the inhibition of macrophage cell death by P5, these results suggest that P5 confers significant protection against lethal sepsis partly by attenuating sepsis-induced tissue injury.

## Discussion

**Figure 8:**
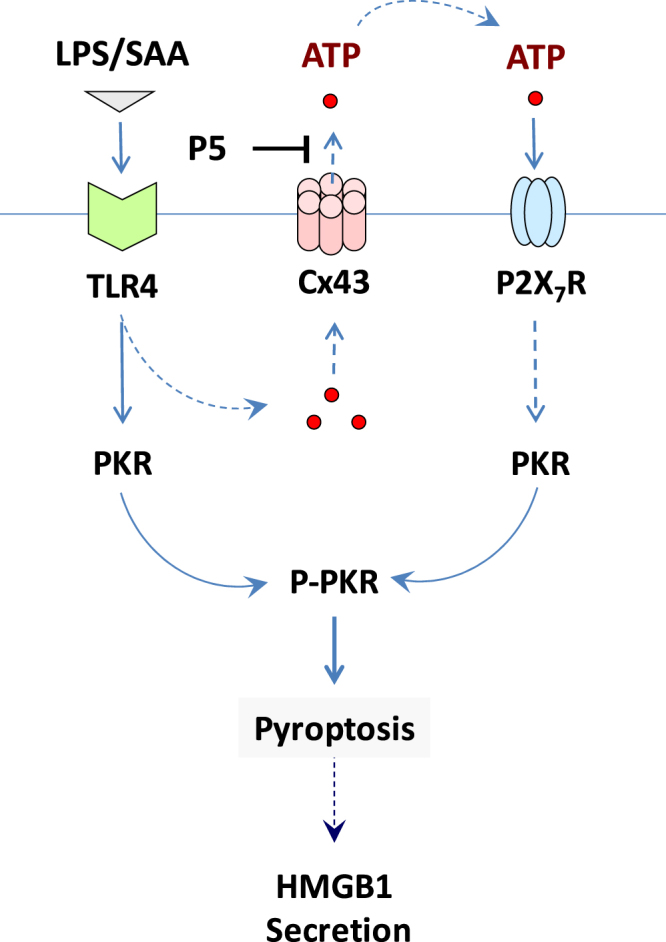
Proposed model for the P5-mediated inhibition of HMGB1 release. Prolonged stimulation with crude LPS or SAA led to the up-regulation of Cx43, which may contribute to extracellular ATP efflux, P2X_7_R-mediated PKR phosphorylation, and resultant PKR-mediated pyroptosis and HMGB1 release. As a novel Cx43 inhibitor, the P5 peptide may block the LPS/SAA-induced ATP efflux, thereby impairing the PKR-mediated HMGB1 release.

Here we reported for the first time that a newly recognized proinflammatory mediator, SAA^9^, resembled the crude endotoxin (*containing bacterial CpG-DNA and lipoproteins*), and up-regulated Cx43 expression and parallel PKR phosphorylation in a TLR4-dependent fashion. In addition, the hexameric Cx43 EL1 mimetic peptide P5 concurrently blocked the LPS-induced macrophage hemichannel activation and resultant ATP and HMGB1 release. These data supports a possibility that the crude LPS- and SAA-induced upregulation of Cx43 expression and hemichannel activities may contribute to the efflux of ATP, which activates the purinergic P2X_7_R to mediate PKR phosphorylation and HMGB1 secretion (Fig. 8). This notion is in agreement with several lines of previous findings. First, ultrapure LPS (*free from contaminating bacterial CpG-DNA or lipoproteins*) failed to induce PKR phosphorylation and HMGB1 secretion, unless the initial LPS priming is accompanied by a second stimulus, ATP^10,36^. Second, Cx43 hemichannels provide a temporal mode of ATP release from activated monocytes/macrophages^33,34^, and the hemichannel-mediated ATP efflux contributed to the P2X_7_R-dependent inflammasome activation^37,38^. It is thus plausible that the Cx43 hemichannel-mediated ATP release contributes to the LPS- and SAA-stimulated PKR phosphorylation and HMGB1 release.

Cytoplasmic membrane-bound Cx43 proteins oligomerize into hexameric hemichannels, which can dock with the hemichannels on adjacent cells to form GJ channels to facilitate intercellular communications in various tissues such as the heart, brain, and vasculature^20,22–24^. Because Cx43 GJ and hemichannels often co-exist in plasma membranes, a definitive elucidation of the functions of Cx43 GJ or hemichannels has been quite challenging. First, genetic deletion would eliminate both channels. Secondly, these two types of Cx channels are susceptible to a similar array of chemical blockers. Lately, attempts to selectively target Cx43 extracellular loops by mimetic peptides such as GAP26 or extracellular loop antibodies have only gained partial success due to interruption of GJ formation after prolonged application. Thus, a more selective Cx43 mimetic peptide targeting the intracellular loop was developed by fusing the GAP19 with an N-terminal transactivator of transcription (TAT) tag (YGRKKRRQRRR) that facilitates the internalization of the fusion peptide across the cytoplasmic membrane. This TAT-GAP19 peptide might interact with the last 10 amino acids of the carboxyl tail of Cx43 to interrupt the interaction between the cytoplasmic loop with the C-terminal tail, thereby selectively blocking Cx43 hemichannels activities^28–30^. Surprisingly, we discovered that GAP26 and TAT-GAP19 divergently affected macrophage hemichannel activities *in vitro*, and differently affected the outcomes of lethal sepsis *in vivo*. These intriguing findings further supported a possible role of macrophage Cx43 hemichannel in the pathogenesis of lethal systemic inflammation.

In innate immune cells such as macrophages, Cx43 exists predominantly in the form of hemichannels that connect cell interior to extracellular milieu^24,26,39^. Likewise, macrophages may also use Panx1 hemichannels to mediate the efflux of ATP^40^, which activates P2X_7_R to trigger subsequent inflammasome activation and pyroptosis^40^. Thus, the definitive delineation of macrophage Cx43 or Panx1 hemichannel functions has also been challenging. Thus, we designed a panel of smaller peptides based on the most conserved sequence of the Cx43 EL1, and screened each for attenuating macrophage hemichannel activities. Consistent with the recognized functional significance of cysteine (Cys) residue in the EL1, one of the Cys-containing peptide, P5, effectively attenuated the endotoxin-induced elevation of macrophage hemichannel activities. Because the P5 peptide did not alter macrophage LY uptake in calcium-containing medium, it unlikely cross interferes Panx1 hemichannel permeability. Furthermore, it did not affect the Cx43 GJ formation or communication in non-immune cells, suggesting P5 as a possible selective blocker of macrophage Cx43 hemichannel activities without compromising GJ communications. Under inflammatory conditions, macrophages also form Cx43-containing GJ channel with cardiomyocytes^41^, epithelial cells^42,43^, and endothelial cells^19^, suggesting an exciting possibility that innate immune cells may communicate with non-immune cells through Cx43-containing GJ channels to orchestrate inflammatory responses^39,44^. Therefore, it will be critically important to investigate whether the P5 peptide or other mimetic peptides confer protection through disrupting inter-cellular communication between immune and non-immune cells.

The mechanism by which P5 peptide selectively blocks macrophage hemichannels remains a subject of future investigation. Its overlapping mimetic peptide, the GAP26, is believed to interact with the extracellular loops, consequently closing hemichannels or preventing gap junction formation by blocking connexon-connexon anchoring. Intriguingly, we did not find evidence of P5 interacting with synthetic full-length Cx43 EL1 or EL2 peptide as judged by the Surface Plasmon Resonance Analysis (data not shown), although a partly overlapping peptide, P4 (QPGCEN), exhibited a highly reproducible binding to the Cx43 EL1 peptide (Kd = 20.0 µM). However, the P5 peptide significantly attenuated the LPS-induced ATP release and trypan blue uptake by macrophage cultures, supporting a possibility that Cx43 may facilitate extracellular ATP efflux to trigger PKR phosphorylation/activation and PKR-mediated pyroptosis (Fig. 8).

As aforementioned, pyroptosis is an inflammatory caspase-dependent form of programmed necrosis occurring during microbial infection. However, distinct caspases (e.g., human caspase-1, -4, -5, -13, and -14, as well as murine caspase-11 and -12) are involved in pyroptosis as opposed to other forms of programmed cell death, such as apoptosis, which is defined as the caspase-2, -3, -6, -7, -8, -9, and -10-mediated cell death. Morphologically, apoptosis is characterized by nuclear condensation, chromatin cleavage, formation of apoptotic bodies, and exposure of surface molecules targeting for phagocytosis. As a measure of apoptosis, the fragmentation of chromosomal DNA can be biochemically detected by using a highly sensitive TUNEL method. Nevertheless, recent studies have suggested that pyroptotic cells also undergo DNA fragmentation and, like apoptotic cells, similarly show positive TUNEL staining^45,46^.

Unlike apoptosis, pyroptosis is characterized by both cell swelling and loss of plasma membrane integrity that precipitates the release of cytoplasmic content (e.g., HMGB1) into the extracellular milieu. This feature is shared with necrosis, which is manifested biochemically as the release of cytosolic enzymes (including LDH, Fig. 7a) and the uptake of membrane-impermeant trypan blue dye (Fig. 5c). Thus, the loss of structural integrity of the plasma membrane might be a hallmark of both pyroptosis and necrosis. In light of the important role of PKR in the regulation of Caspase-1-dependent pyroptosis^11^ and the receptor interacting protein (RIP)1/RIP3-dependent programmed necrosis (necroptosis)^47^, it will be important to determine whether P5 inhibits the LPS-induced HMGB1 release and/or sepsis-induced lung injury partly through impairing PKR-mediated pyroptosis and/or necroptosis.

Regardless, the P5 peptide dose-dependently attenuated the LPS-induced release of HMGB1, a critically important mediator of infection- and injury-elicited inflammatory diseases^6,13–15^. Consistently, we found that P5 peptide conferred a significant protection against both I/R injury and lethal microbial infection. Our findings were in agreement with the well-documented role of Cx43 in the pathogenesis of cardiac and brain I/R injury^20^, but further suggested the possible involvement of Cx43 hemichannel in sterile injury. Because global Cx43 knockout mice could not survive after birth due to a possible failure of pulmonary function^48^, conditional knockout of Cx43 in myeloid cells might facilitate the understanding of Cx43 hemichannels in injurious and infectious inflammatory diseases.

In summary, here we report that a newly identified proinflammatory mediator, SAA, resembled bacterial endotoxin by stimulating macrophages to parallelly up-regulate Cx43 expression and PKR phosphorylation in a TLR4-dependent fashion. Different Cx43 mimetic peptides, the GAP26 and TAT-GAP19, distinctly affected the endotoxin-induced elevation of macrophage hemichannel activities *in vitro*, and divergently altered the outcome of lethal sepsis *in vivo*. A cysteine-containing hexamer peptide, P5, attenuated the endotoxin-induced elevation of Cx43 hemichannel activities without impairing the Cx43 GJ formation and function. *In vitro*, the P5 peptide significantly attenuated the endotoxin-induced uptake of trypan blue and release of ATP and HMGB1 by macrophage cultures. *In vivo*, the P5 peptide conferred significant protection against both I/R injury and lethal microbial infections. Collectively, these findings have suggested a pathogenic role of Cx43 hemichannel in injury- and infection-elicited inflammation possibly through facilitating the extracellular ATP efflux to trigger PKR phosphorylation/activation.

## Material and Methods

### Materials

Bacterial endotoxin (lipopolysaccharide, LPS, *E. coli 0111:B4*), Lucifer Yellow (L0144), paraformaldehyde (P6148) and mouse anti-β-actin antibodies (A1978) were obtained from Sigma-Aldrich (St. Louis, MO, USA). Recombinant human SAA (also called Apo-SAA, *Cat. No*. 300-13) was obtained from PeproTech (Rocky Hill, NJ). The apo-SAA is almost identical to human Apo-SAA1α, except for the presence of an N-terminal methionine, the substitution of asparagine for aspartic acid at position 60, and arginine for histidine at position 71, the latter two substituted residues are present in Apo-SAA2β. GAP26 (VCYDKSFPISHVR), TAT-GAP19 (YGRKKRRQRRRK-QIEIKKFK) and P5 (ENVCYD) were synthesized by Genscript (Piscataway, NJ). To characterize the mimetic peptide, the mass spectrometry was carried out using the Thermo Deca XP Ion Trap Mass Spectrometer as previously described^49^. Dulbecco’s modified Eagle medium (DMEM, 11995-065) and penicillin/streptomycin (cat. 15140-122) were from Invitrogen/Life Technologies (Carlsbad, CA). Fetal bovine serum was from Crystalgen (FBS-500, Commack, NY) and heat-inactivated before use. Anti-HMGB1 antibody was affinity-purified from serum of rat HMGB1-immunized rabbits as previously described^6^. HRP conjugated goat anti-mouse IgG and donkey anti-rabbit IgG were from Santa Cruz Biotechnology Inc. (sc-2060, Dallas) and GE Healthcare (NA934; Port Washington, NY), respectively. Anti-phospho-PKR antibody (Thr^451^, 07–886) was from Millipore. An affinity-purified polyclonal anti-Cx43 antibody was generated in rabbits as previously described^50^^.^ TLR2 and TLR4 KO mice on a C57BL/6 genetic background were maintained at The Feinstein Institute for Medical Research as previously described^9,50^. Because the KO mice were derived from C57BL/6 mice, small colonies of wild-type C57BL/6 (Jackson Laboratory) were maintained under the same conditions. Balb/c male mice (7–8 wks old, 20–25 g) were obtained from Taconic Biosciences (Hudson, NY). Macrophage cell line RAW 264.7 and NIH 3T3 fibroblasts were obtained from American Type Culture Collection (ATCC, Rockville, MD).

### Cell culture

Primary peritoneal macrophages were isolated from male Balb/c or C57BL/6 mice (7–8 wks, 20–25 g) at 2–3 days after intraperitoneal injection of 2 ml thioglycollate broth (4%) as previously described^51,52^. Briefly, mice were sacrificed by CO_2_ asphyxiation, and the abdomen region was cleaned with 70% ethanol before making a small excision of the abdominal skin to expose the abdominal wall, and to insert a catheter into viscera-free pocket in order to wash out peritoneal macrophages with 7.0 ml of 11.6% sucrose solution. Macrophages were cultured in DMEM supplemented with 1% penicillin/streptomycin and 10% FBS. When reaching 70–80% confluence, adherent cells were gently washed with, and cultured in, OPTI-MEM I before stimulating with crude LPS, purified recombinant HMGB1 or SAA, in the absence or presence of Cx43 mimetic peptide. The cellular levels of Cx43 and phosphorylated PKR, as well as the extracellular release of HMGB1 and other cytokines/chemokines were determined by Western blotting analysis and Cytokine Antibody Arrays as previous described^9^.

To elucidate the mechanisms underlying the regulation of hemichannel expression, we determined whether the disruption of TLR2 or TLR4 expression led to impairment of LPS- or SAA-induced upregulation of Cx43 proteins. Primary peritoneal macrophages was isolated from wild-type, TLR2- or TLR4-deficient mice, and stimulated with crude LPS, purified HMGB1 or SAA, for 16 h, and the levels of Cx43 and phosphor-PKR in activated macrophages were measured by Western blotting.

### Western blotting

The cellular levels of Cx43 and phosphor-PKR were determined by Western blotting analysis using an affinity-purified rabbit anti-Cx43 or anti-phospho-PKR (Thr^451^) IgGs as previously described^9,25,53^. The levels of HMGB1 in the culture medium were determined by Western blotting analysis as described previously^9^. The level of β-actin was used as a reference of total cellular proteins. Briefly, an equal volume of culture medium (conditioned by identical macrophage cell numbers) was harvested, and protein content was concentrated by ultrafiltration (with a molecular weight cutoff of 10.0 kDa), and then normalized to the same volume with a sample buffer. Proteins in equal sample volume were resolved on sodium dodecyl sulfate (SDS)-polyacrylamide gels, and transferred to polyvinylidene difluoride (PVDF) membranes. After blocking with 5% nonfat milk, the membrane was incubated with respective antibodies (anti-Cx43, 1:1000; anti-phospho-PKR, 1:1000; anti-HMGB1, 1:1,000; anti-β-actin. 1:5,000) overnight. Subsequently, the membrane was incubated with the appropriate secondary antibody, and the immunoreactive bands were visualized by chemiluminescence techniques.

### Cx43 immunostaining

Primary peritoneal macrophages were isolated from Balb/c mice, stimulated with LPS or SAA for 6 h, and immunostained with an affinity-purified rabbit anti-Cx43 IgGs (1:1,000 dilution at 4 °C) as previously described^53^. Non-immune NIH 3T3 cells were grown on glass coverslips to confluency to allow formation of gap junctional channels. The growth medium was changed to DMEM before an overnight incubation with P5 (20 µM). Cells were then fixed with 2% paraformaldehyde, incubated for 16 hr with a well-characterized affinity-purified rabbit anti-Cx43 IgGs as previously described^53^. After extensive washing, cells were incubated with donkey anti-rabbit Alexa 488 (Invitrogen) for 1 hr at room temperature. The coverslips were subsequently mounted on glass slides using anti-fade medium and examined using an epi-fluorescence microscope.

### Gap junction channel and hemichannel permeability assays

Because connexon is the structural unit for both hemichannel (1 connexon) and gap junction (2 connexons from opposing cells), which are both permeable to similar molecular weight molecules, such as the Lucifer yellow (LY). Thus, the LY dye uptake could be used to determine the channel permeability of both hemichannel (as indicated by the rate of LY entry into the cells) and gap junction channels (as judged by the distance of intercellular LY diffusion). The LY dye uptake assay relied on the ability of this dye present in the extracellular milieu to cross the plasma membrane and became concentrated inside of the cell, which could be quantified by fluorescence microscopy. To determine the effects of Cx43 mimetic peptides on hemichannel permeability, RAW 264.7 cells were subjected to LPS stimulation (0.5 µg/ml, 16 h) before replacing the medium with fresh DMEM containing various Cx43 mimetic peptides, GAP26 (100 µM), TAT-GAP19 (200 µM) or P5 (20 µM). Following a brief incubation (15 min), EGTA (1 mM) was added to deplete free calcium prior to the addition of LY dye (1%). Fifteen min later, cells were fixed with 2% paraformaldehyde, and the number of LY-positive cells was counted. The ratio of LY-positive cells vs. total cell counts (DAPI staining) was used as the measurement of hemichannel permeability.

We used NIH 3T3 fibroblasts to investigate the effects of Cx43 mimetic peptides on GJ permeability employing a technique termed scrape-loading. After overnight or 20 min incubation with or without Cx43 mimetic peptides in DMEM, EGTA was added to DMEM to deplete free calcium. Immediately afterwards, 1% LY and 1% rhodamine dextran was added into PBS, followed by scraping the cell monolayer with a pipette tip loading tracers into cytoplasmic compartment of scraped cells. Tracers were allowed to diffuse to adjacent cells for 2 min before immobilized by PF fixation for 10 minutes. Cells were subsequently extensively washed in PBS three times and coversliped to allow examination of LY diffusion.

### ATP release assay

In addition to the LY dye uptake, we also employed the ATP release assay to measure the Cx43 hemichannel activities. The current gold standard for ATP quantification relied on luciferin:luciferase chemistry where the enzyme luciferase catalyzed the oxidation of ATP and luciferin ultimately resulting in the generation of a photon which could be quantified by luminometry using a luminometer plate reader (Synergy H1 Hybrid, Biotek, Winooski, U.S.A.) and the amount of ATP was calculated from standard curve. Briefly, RAW 264.7 cells were cultured in serum-free DMEM medium, and stimulated with LPS (1 µg/ml) in the absence or presence of Cx43 mimetic peptide (P5, 20 µM). The cell-conditioned culture medium was collected and subjected to ATP measurement. We used the Molecular Probes® ATP Determination Kit (Cat. # A22066, Life Technologies), which was based on luciferase’s absolute requirement for ATP in producing light (emission maximum ~560 nm at pH 7.8), to measure the extracellular ATP levels in LPS-stimulated RAW 264.7 cells.

### Trypan blue uptake assay

Cell viability was evaluated by the trypan blue exclusion method, which distinguished the unstained viable cells from nonviable cells that taken up the dye to exhibit a distinctive blue color. Specifically, murine macrophage-like RAW 264.7 cells were stimulated with LPS (1.0 µg/ml) in the absence or presence of P5 peptide (10 µg/ml) for 16 h, and the culture medium was then replaced with 1 × PBS containing 0.08% trypan blue (Cat. # 15250-061, Invitrogen). Phase contrast images of multiple fields were randomly captured, and the percentage of trypan blue-stained cells was calculated.

### Cytokine antibody array

Murine Cytokine Antibody Arrays (Cat. No. M0308003, RayBiotech Inc., Norcross, GA, USA), which respectively detect 62 cytokines on one membrane, were used to determine cytokine levels in macrophage-conditioned culture medium as previously described^51,54^. Briefly, the membranes were sequentially incubated with equal volumes cell-conditioned culture medium (200 μl), primary biotin-conjugated antibodies, and horseradish peroxidase–conjugated streptavidin.

### Animal model of polymicrobial sepsis and hepatic I/R injury

This study was approved and performed in accordance with the guidelines of the Institutional Animal Care and Use Committee of the Feinstein Institute for Medical Research, Manhasset, New York, USA (Animal Protocol #2008-033, Approval date: September 10, 2014). To evaluate the effect of various Cx43 mimetic peptides on sepsis lethality, a clinically relevant animal model of sepsis induced by cecal ligation and puncture (CLP) was employed. Briefly, the cecum of Balb/c mice was ligated at about 5 mm from the cecal tip, and then punctured once with a 22-gauge needle. GAP26, TAT-GAP19 or P5 peptides were administered intraperitoneally into mice at indicated doses and time points, and animal survival rates were monitored for up to 2 wks. The Kaplan-Meier method was used to compare the differences in mortality rates between groups.

Male C57BL/6 mice (20–25 g) were subjected to hepatic ischemia/reperfusion by temporally clamping the hepatic artery and portal vein for 60 min, which typically produced ischemia in 70% of the liver. At the beginning of the reperfusion, 0.2 ml saline, P5 (10.0 mg/kg BW) was injected via the internal jugular vein. At 24 h after the onset of ischemia, animals were euthanized to harvest blood to measure serum levels of hepatic injury markers such as alanine aminotransferase (ALT) and aspartate aminotransferase (AST) using commercial kits.

### TUNEL staining

Upper and lower lobe lung tissues were collected at 24 h post CLP, fixed in 4% paraformaldehyde, embedded in paraffin, and cut into 5 µm thick sections. After a digestion with proteinase K (20 µg/ml, Cat# ST532, Beyotime Biotech, Shanghai, China) at 37 °C for 23 min, sections were stained using a terminal deoxynucleotidyl transferase dUTP nick-end labeling (TUNEL) kit (One-step TUNEL staining kit, Cat# C1086, Beyotime Biotech, Shanghai, China), and counterstained with DAPI to visualize all cells. Five to eight fields of view were randomly selected for each tissue section, and the number of TUNEL-positive green cells was expressed as an average of percentage of total DAPI-positive blue cells.

### Statistical analysis

Data are expressed as mean ± SEM of at least two independent experiments in triplicates (n = 2). Student’s t-test was used for comparison between two groups. One-way analyses of variance (ANOVA) followed by the Tukey test for multiple comparisons were used to compare between different groups. The Kaplan-Meier method was used to compare the differences in mortality rates between groups. A *P* value < 0.05 was considered statistically significant.

## Electronic supplementary material

Supplementary Information
