# Supplementary material for: Connexin 43 Hemichannel as a Novel Mediator of Sterile and Infectious Inflammatory Diseases
Source: Sci Rep. 2018 Jan 9;8:166. doi: 10.1038/s41598-017-18452-1 (PMC5760527; doi:10.1038/s41598-017-18452-1)
Supplement: Supplementary file 1 — Supplementary Information [file 41598_2017_18452_MOESM1_ESM.doc]

**Connexin 43 Hemichannel as a Novel Mediator of Sterile and Infectious Inflammatory Diseases.**

Wei Li ^1,2,3 *^, Guoqiang Bao ^2, 4^, Weiqiang Chen ^1,2^, Xiaoling Qiang ^1,2^, Shu Zhu ^1,2^, Shuaiwei Wang ^3^, Mingzhu He ^2^, Gaifeng Ma ^2^, Mahendar Ochani ^2^, Yousef Al-Abed ^2^, Huan Yang ^2^, Kevin J. Tracey ^2^, Ping Wang ^2^, John D’Angelo ^1^, and Haichao Wang ^1,2,*^

**^1^** Department of Emergency Medicine, North Shore University Hospital, Northwell Health, Manhasset, NY 11030, USA; **^2^** The Feinstein Institute for Medical Research, 350 Community Drive, Manhasset, NY 11030, USA; ^3^ International Laboratory of Sepsis Research, Huaihe Hospital, Henan University, Kaifeng, Henan, 475000, China; ^4^ Department of General Surgery, Tangdu Hospital, The 4^th^ Military Medical University, Xi'an, Shaanxi, 710032, China;

**Running title:** Therapeutic modulation of Cx43 hemichannel activities.

**Figure S1. Exogenous bacterial endotoxin (LPS) and endogenous inflammatory cytokines (SAA) up-regulated Cx43 expression and PKR phosphorylation in macrophages.** Primary murine peritoneal macrophages were isolated from wild-type or TLR4-deficient C57BL/6 mice, and stimulated with crude LPS, recombinant HMGB1, or SAA for 16 h. The cellular levels of Cx43 and phospho-PKR (P-PKR) were measured by Western blotting.

**Figure S2. P5 peptide selectively inhibited endotoxin-induced HMGB1 release**. RAW 264.7 cells were stimulated with LPS for 16 h in the absence or presence of P5 peptide at indicated concentrations, and the levels of HMGB1 in the macrophage-conditioned culture medium were determined by Western blotting analysis. Shown above were two representative full-length Western blots.
